# Genetic transformation of GmFBX322 gene and salt tolerance physiology in soybean

**DOI:** 10.1371/journal.pone.0307706

**Published:** 2024-09-12

**Authors:** Hongli He, Yuhan Zhang, Shengli Xu, Xingzheng Zhang, Xiangdong Yang, Yunqing Cheng

**Affiliations:** 1 College of Life Sciences, Jilin Normal University, Siping, China; 2 College of Life Sciences, Jilin Academy of Agricultural Sciences, Changchun, China; Assam Agricultural University Faculty of Agriculture, INDIA

## Abstract

Soybean is one of the most important food crops, breeding salt-tolerant soybean varieties is of great significance to alleviate soybean shortage. In this study, the F-box protein family homologous gene GmFBX322 was cloned from the soybean variety Williams 82 and overexpressed in the Shennong 9 soybean variety to further study and explore the physiological mechanism of soybean salt tolerance. GmFBX322 was constructed on the vector pTF101:35S, and integrated into the genome of Shennong 9 soybean variety by Agrobacterium EHA101-mediated cotyledonary node transformation technology, and 4 overexpressed transgenic lines were obtained, molecular assays were performed on the transformed plants. The expression of GmFBX322 was detected by qRT-PCR and it was found that the leaves of the 4 transgenic lines increased by 2.49, 2.46, 2.77, 2.95 times compared with the wild type; after salt treatment for 12 hours, it was found that the expression of wild type Shennong 9 Inducible expression of GmFBX322. After 72 hours of salt treatment, the leaves of wild-type Shennong 9 soybean plants showed obvious wilting and chlorosis, while the leaves of GmFBX322 plants overexpressing GmFBX322 showed no obvious changes. The leaves were taken at 0, 6, 12, 24, and 48 hours of salt stress to determine the antioxidant activity. Ability and osmotic adjustment level, etc. The results showed that the catalase activity in the leaves of the transgenic lines 2265, 2267, 2269, and 2271 was 2.47, 2.53, 3.59, 2.96 times that of the wild-type plant after 48 hours of salt treatment; the soluble sugar content was 1.22, 1.14, and 1.22 of the wild-type plant. 1.14, 1.57 times; the proline content is 2.20, 1.83, 1.65, 1.84 times of the wild type. After comparing the physiological indicators determined by the experiment, the transgenic lines performed better than the control group, indicating that overexpression of *GmFBX322* can enhance the salt tolerance of soybean plants. To verify the function of *GmFBX322* gene related to stress resistance, add it to the candidate gene of stress resistance, and provide scientific basis for the selection and breeding of salt-tolerant varieties.

## Introduction

The F-box gene family is one of the largest gene families in plants, and the F-box protein can be abbreviated as FBX, which is so large that it is divided into different subfamilies [1]. The protein encoded by the F-box gene can regulate a variety of life activities, such as regulating plant leaf size, drought and salt stresses, and responding to biological stresses [2]. Studies have shown that the ectopic expression of ZmFBX92 in Arabidopsis thaliana can lead to the enlargement of Arabidopsis leaves, and the opposite effect is observed after reducing the expression level of AtFBX92. This is mainly due to the fact that the F-box protein FBX92 affects cell division, resulting in different cell numbers and ultimately affecting leaf size. At the same time, many of the F-box genes involved in plant drought resistance or salinity resistance also affect the drought resistance and salinity resistance of plants by directly or indirectly affecting plant hormone signaling pathways [3, 4]. For example, the cloning of GmFBX176, a protein encoding gene in soybean F-box, found that GmFBX176 affected the transcription level of stress response genes and regulated ABA-mediated drought and salt stress responses [3]. Finally, the F-box gene can also regulate the closure of stomata to achieve defense against biological stresses, for example, the Arabidopsis F-box protein MAX2 can improve the resistance to bacteria, on the contrary, the stomatal conductance of the MAX2 mutant plants is enhanced, and the stomatal closure is impaired, resulting in pathogen infection [1]. It can be seen that F-box protein plays a wide and important role in plants, and the involvement of F-box protein in the physiological processes related to plant disease resistance and stress resistance has been paid more and more attention [4].

With the intensification of the global greenhouse effect, the frequency of drought disasters in most parts of the world is shortened, the degree of damage is aggravated, and it shows a trend of normalization [5, 6]. The new soybean varieties with higher yield and stronger stress resistance cultivated by transgenic technology are of great significance to solve the problem of soybean shortage. Transgenic technology can improve the ability of soybean plants to withstand drought, heat, alkali and salt. In alkaline environment, a large number of sodium ions in the soil invade cells, enzyme activity in the embryo is reduced, protein metabolism is weakened, hormone regulation is disturbed, the utilization rate of seed reserve nutrients is decreased, and the embryo stops developing or dies directly [7]. High salt also reduces plant water potential. With the increase of salt concentration in culture environment, the free water content in plant leaves decreases, the osmotic potential and pressure potential of plant cells decrease, and the stomatal conductance of plant leaves decreases, thus reducing the transpiration rate [8, 9]. High salt reduced the transport and utilization efficiency of photosynthesis and slowed down the cell respiration rate. High salt disrupts the synthesis of proteins and lipids in plants, and at the same time, interferes with the synthesis of chlorophyll and carotenoids, making the leaves green [10]. Research shows high salinity has different effects on the growth and development of plants.

The F-box gene was found in a variety of sequencing plants, of which 509 were identified in soybean [11]. It is reported that F-box gene plays an important role in mediating various types of stress. In this study, the overexpression vector of GmFBX322 gene in F-box family was introduced into Shenong 9 soybean variety to obtain stable genetic lines. The physiological indexes of salt tolerance were tested, and the overexpression of GmFBX322 could improve the tolerance of soybean to salt stress. To verify the function of GmFBX322 gene related to stress resistance, and to include it as a candidate gene for stress resistance, so as to provide candidate gene elements for subsequent soybean auxiliary breeding, improve the speed of breeding excellent varieties, and provide scientific basis for the cultivation of new salt-tolerant varieties.

## Materials and methods

### Preparation of engineered strains

The RNA of Williams 82 soybean leaves, 4 μL of RNA template, 8 μL of RNase H2O, 4 μL of Eraser Mix 4 × gDNA, 5 μL of MasterMixII × 4 μL of TRUERT4 μL were used as materials for reverse transcription, and finally soybean RNA was used as template to synthesize cDNA. The PCR50 system of the synthesized gene of interest was amplified at 94°C for 5 min, 94° C for 30 s, 57° C for 30 s, 72° Cfor 60 s, 35 cycles, and finally extended for 10 min. The primersare:BAR-F5’-ATGGAGTTGCAAGATTTGCC-3’. BAR-R5’-GACATGTT TAGGCCTAATTTCTATT-3’. Digestion vector pTF101-35S with restriction endonuclease Xba I. A 25uL reaction was prepared with 1 μL of XbaI, 2 μL of 10×Buffer, 2 μL of 0.1% BSA, 5 μL of pTF101-35S, and finally 10 μL of ddH2O. The reaction temperature is 37°C, the reaction time should be 1h, and the carrier is recovered after the experiment.

The recombinant vector was transformed into E. coli, and the recombinant vector was transferred to E. coli by heat shock. Colony PCR positive clone detection and gene sequencing First, 1/3 of a single colony was mixed with 5 μL of sterile water by a sterile pipette tip, and 1 μL of the mixture was added to the PCR (10uL) system with upstream and downstream primers, and then the positive clones were identified by agarose gel electrophoresis band position. PCR reaction conditions: 94° C for 5 min, 94°C for 30 s, 57°C for 60 s, 72° C for 30 cycles, and finally extended for 10 min, in which the primers were: G9-Q-F: 5’-CTCTCAGACCATCCACACCAT-3’, G9-R:5’-GCTTGTCCACTCCCAATGT T-3’. Positive clonal single colonies were selected and propagated with LB liquid medium containing antibiotics. 100 μL of the propagated bacterial solution was piped for sequencing, and the sequence analysis was performed with the universal primers of the vector. The specific step of transforming Agrobacterium into GmFBX322 overexpression vector is to activate E. coli with correct sequencing results and propagate. The plasmid was extracted by lysis method, Agrobacterium competent cells were prepared, and the recombinant plasmid was transferred into Agrobacterium competent cells by freeze-thaw method. Agrobacterium positive clone detection first picked 1/3 of a single colony and added it to the PCR system with upstream and downstream primers, and the PCR product was identified by the band size displayed by 1.5% agarose gel electrophoresis to identify whether it was a positive clone. The strip showed the correct colony propagation and was used for bacterial protection. The primers are:G9-Q-F:5’-CTCTCAGACCATCCCACCAT-3’, G9-R:5’-GCTTGTCCACTCC CAATGTT-3’, Reaction conditions: PCR reaction conditions: 94°C for 5 min, 94° C for 30 s, 57°C for 60 s, 72° C for 30 cycles, and finally extended for 10 min.

### Preparation of transgenic line(s)

GmFBX322, the homology gene of the F-box protein family, was cloned from the soybean variety Will iams 82, and GmFBX322 was constructed on the vector pTF101:35S, which was integrated into the genome of Shennong 9 soybean variety through Agrobacterium EHA101-mediated cotyledon transformation technology.

### Testing of transgenic line(s)

In general, in the experiment, we take three methods to carry out positive detection herbicide screening. In the early flowering stage of soybean, three compound leaves of different plants in the same position were selected to apply glufosinate-ammonium herbicide at a working concentration of 3 ‰, and the condition of soybean leaves was observed and recorded after 3 days.

The first one takes a small amount of soybean leaves in a centrifuge tube, adds sterile water and grinds them with a grinding rod, and uses a BAR test strip for detection.

The second GmFBX322 gene detection using soybean DNA: PCR, PCR reaction conditions: 94° C for 5 min, 94°C for 30 s, 57°C for 60 s, 72° C for 30 cycles, and finally extended for 10 min, primers for G9 series soybean DNA BAR gene detection: BAR-F: 5’- CAGCTGCCAGAAACCCACGT-3’, BAR-R: 5’- CTGCACCATCGTCAACCACT-3’, reaction conditions: 94° C for 5 min, 94° C for 30 s, 58°C for 60 s, 72°C for 30 s for 28 cycles of final extension.

The third method was to detect the gene expression level of soybean GmFBX322, germinate at 23°C in the dark, and then transfer to Hogland complete nutrient solution after 4 days, and the culture conditions were the same as above, and the culture was incubated for 10 days. Seedlings with consistent growth were selected, and some of them were continued and the other part was cultured with Hogeland complete nutrient solution containing 150 mM NaCl under unchanged conditions. After 24 h, 0.1 g of fully unfolded compound leaves were taken from each soybean plant in a 1.5 mL centrifuge tube, quickly liquid nitrogen, and stored in an ultra-low temperature freezer for later use. Soybean RNA was used as a template to synthesize cDNA. qRT-PCR validation method: The expression of GmFBX322 gene was analyzed in an RT-PCR (Mx 3000p) quantitative PCR instrument. The soybean Actin gene was used as the internal reference gene, and the primers Actin-F: 5’-GAGCTATGAATTGCCTGATGG-3’ and Actin-R: 5’-CGTTTCATGAATTCCAGTAGC-3’ were primers. Reaction conditions: 95°C for 1 min, 95°C for 5 s, 60°C for 10 s, 72°C for 30 s, 40 cycles, and then extended for 10 min. Perform at least 3 technical replicates of each pair of primers to obtain confident experimental results.

### Analysis of salt tolerance in soybeans

Firstly, the soybean seeds germinated at 23°C in the dark, and then transferred to Hogaland complete nutrient solution after 4 days, and the culture conditions were 23°C, 16 h light, 8 h dark, and the second compound leaf was fully expanded (about 10 days) [12, 13]. Plants with the same growth were selected and continued with Hogland’s complete nutrient solution and Hogrande complete nutrient solution containing 150 mM NaCl under unchanged cultural conditions [13]. Observe the growth of the plants every day and take photos to record them.

Secondly, the physiological indexes of salt tolerance of soybean plants were determined, and three kinds of measurements were carried out.

In the first case, CAT activity was measured, and the catalase activity of soybean leaves was measur7ed at five time points (sampling immediately after treatment), 6, 12, 24, and 48 h [13].

In the second case, soluble sugar was detected, and soybean leaves were taken to measure the soluble sugar content by anthone method at five time points of culture 0 (sampling immediately after treatment), 6, 12, 24, and 48 h. and draw a standard curve [14].

In the third method, the proline content was determined, and the soybean leaves were taken after the 0th (sampling immediately after treatment), 6, 12, 24, and 48 h of culture, and the acid ninhydrin method was used to detect the proline content. Draw a standard curve [14].

### Statistics and analysis

The data collected in this study were processed and analyzed using EXCEL 2007 and IMB SPSS Statistics 22. EXCEL 2007 plotted graphs and standard error bars were calculated using the T-test, and SPSS Statistics 22 analysis data were analyzed using the LSD test to calculate the significance level of the difference (there were two significant levels of 0.05 and 0.01; the confidence interval was 95%), and 3 biological replicates.

## Results

### IBioinformatics analysis of GmFBXs

A heat map of the differential transcript abundance of these19 F-box genes in the 3 tissue types shows that most F-box genes have broad expression patterns in soybean. The gene expression of GmFBX322 was significantly higher than that of other genes.

**Fig 1:**
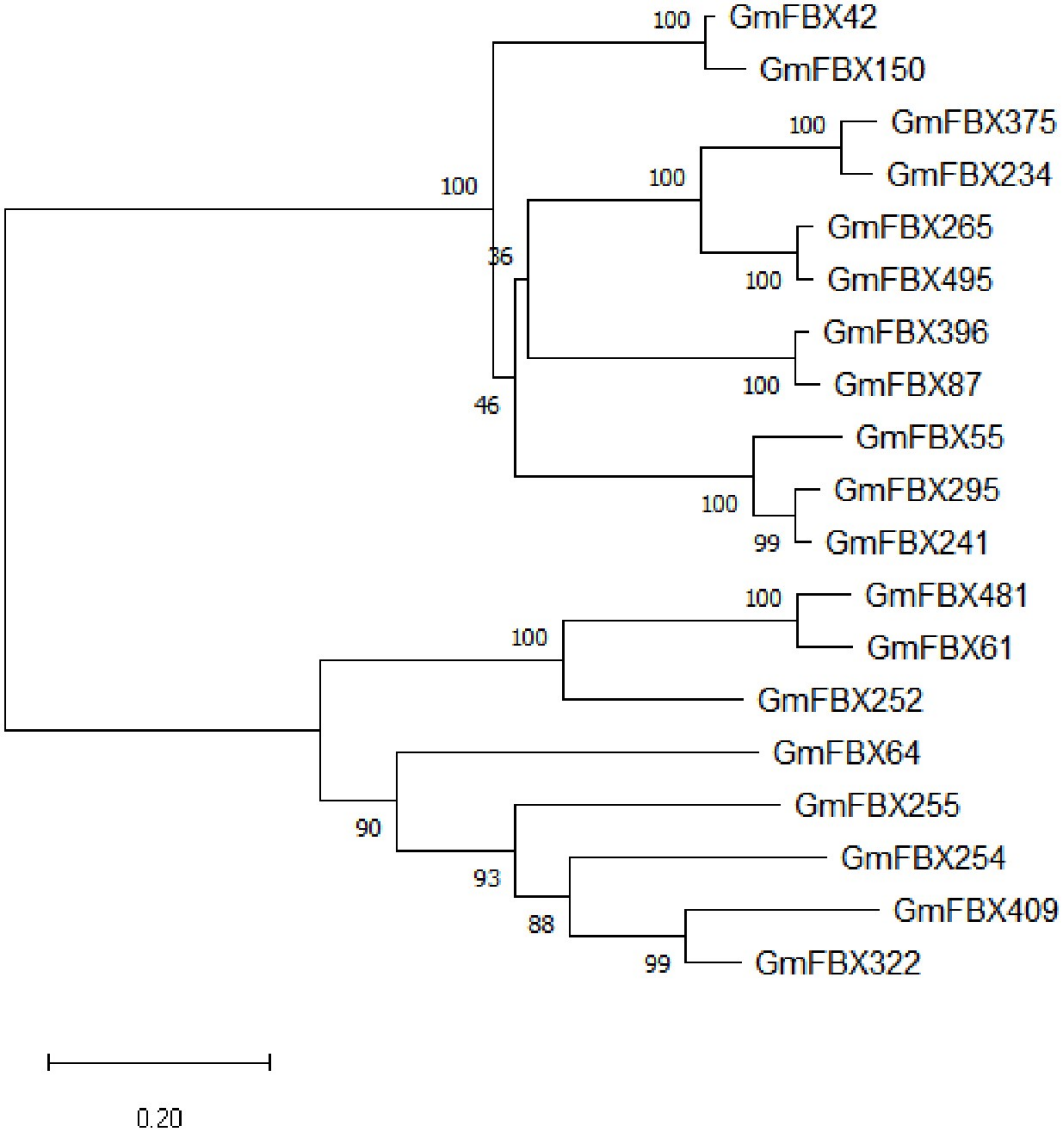
The evolutionary history was inferred using the Neighbor-Joining method. The optimal tree is shown. The percentage of replicate trees in which the associated taxa clustered together in the bootstrap test (1000 replicates) are shown next to the branches. The evolutionary distances were computed using the Poisson correction method and are in the units of the number of amino acid substitutions per site. This analysis involved 19 amino acid sequences. All ambiguous positions were removed for each sequence pair. There were a total of 388 positions in the final dataset. Evolutionary analyses were conducted in MEGA11.

The adjacent joining method was used to construct the phylogenetic tree by using Mega-11software (Fig 1). The results showed that the soybean FBP family protein was divided into two groups, GmFBX322 was in group 2, and GmFBX409 was the most closely related.

### Agrobacterium mediates the genetic transformation of soybean cotyledons

**Fig 2:**
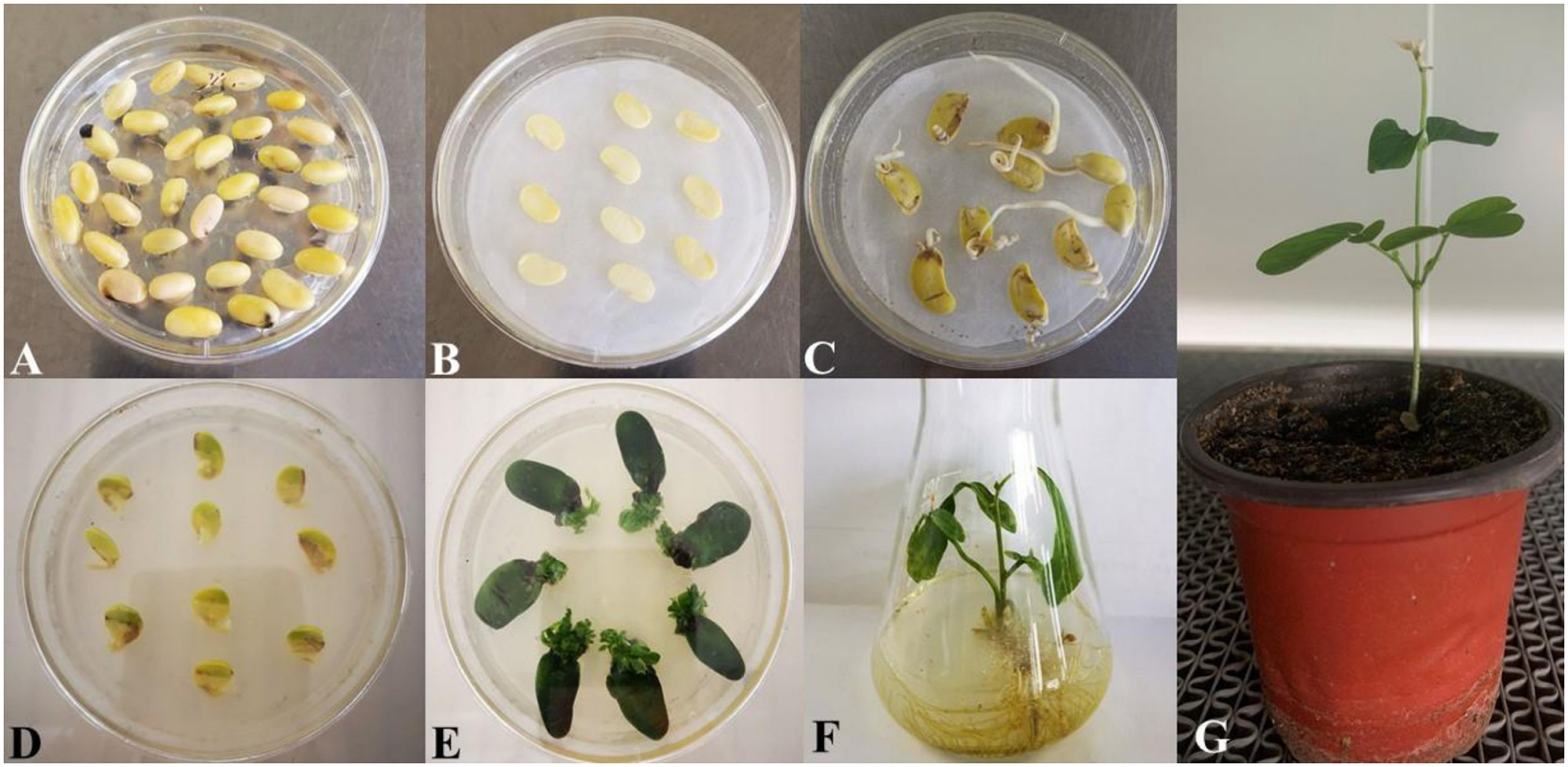
Agrobacterium-mediated genetic transformation of soybean cotyledon nodes. (A)Seed germination. (B)The seeds are further impregnated. (c)coculture. (D)Induce budding. (E)Induce bud elongation. (F)Induce rooting. (G)Transplant the rooted plants into pots.

Mature soybean seeds with full grains and clean surfaces were selected and laid in a single layer on a petri dish, with 100 seeds per dish. The petri dish was placed in a desiccator. Next, 5 mL of concentrated hydrochloric acid was added to a glass beaker containing 100 mL of sodium hypochlorite, and the beaker was placed in the center of the desiccator. The desiccator lid was tightly closed, and the seam between the lid and the base was sealed further with petroleum jelly. After 16 h, the soybeans were removed, and the mixed liquid was poured into a waste bottle for unified treatment. The seeds were stored in an ultraclean workbench, and aseptic air was blown over them for 5 min to dissipate the chlorine gas. The seeds were then sealed in a plastic wrap. The soybean seeds were evenly planted in germination medium with the umbilicus facing downwards, placing 20–25 seeds per dish. The dishes were sealed with breathable tape and incubated in the dark at 23°C for 24 h to allow the seeds to fully absorb water [15 and 16] (Fig 2A).

A sterile scalpel was used to cut longitudinally along the midline of the soybean, splitting it into two and exposing the cotyledons. The split soybeans were soaked in Agrobacterium solution resuspended in CCM liquid medium and shaken to ensure that the solution fully contacted the cut surfaces, improving transformation efficiency. After 30 min, the soybeans were transferred to CCM solid medium lined with sterilized filter paper, with the cut side facing upward. Ten plates were neatly placed per dish, sealed with breathable tape, and incubated in the dark at 23°C for 5 d (Fig 2B and 2C).

Agrobacterium EHA101, transformed with a plasmid carrying the GmFBX322 gene, was used to infect the explants of the Shennong No. 9 soybean cultivar. The genetic transformation process included seed germination, Agrobacterium infection, coculture of Agrobacterium and soybean explants, bud induction, bud elongation, rooting, seedling refinement, and transplantation to soil (Fig 2C–2G). Twenty transformations were conducted using the EHA101 Agrobacterium-mediated genetic transformation of soybean cotyledons. Out of 1980 infected soybean explants, 32 positive plants were obtained, resulting in a transformation efficiency of about 1.62% [17–19].

### Detection of positive soybean plants overexpressing GmFBX322

After selecting wild-type and transgenic Shennong 9 plants, glufosinate-ammonium solution was applied to three compound leaves at a concentration of 3‰ for 72 h. The results showed that the leaves of wild-type Shennong 9 lost their green color, indicating that they were not resistant to the glufosinate-ammonium herbicide. In contrast, the leaves of the GmFBX322-overexpressing lines showed no change, indicating that these lines were resistant to the glufosinate-ammonium herbicide.

**Fig 3:**
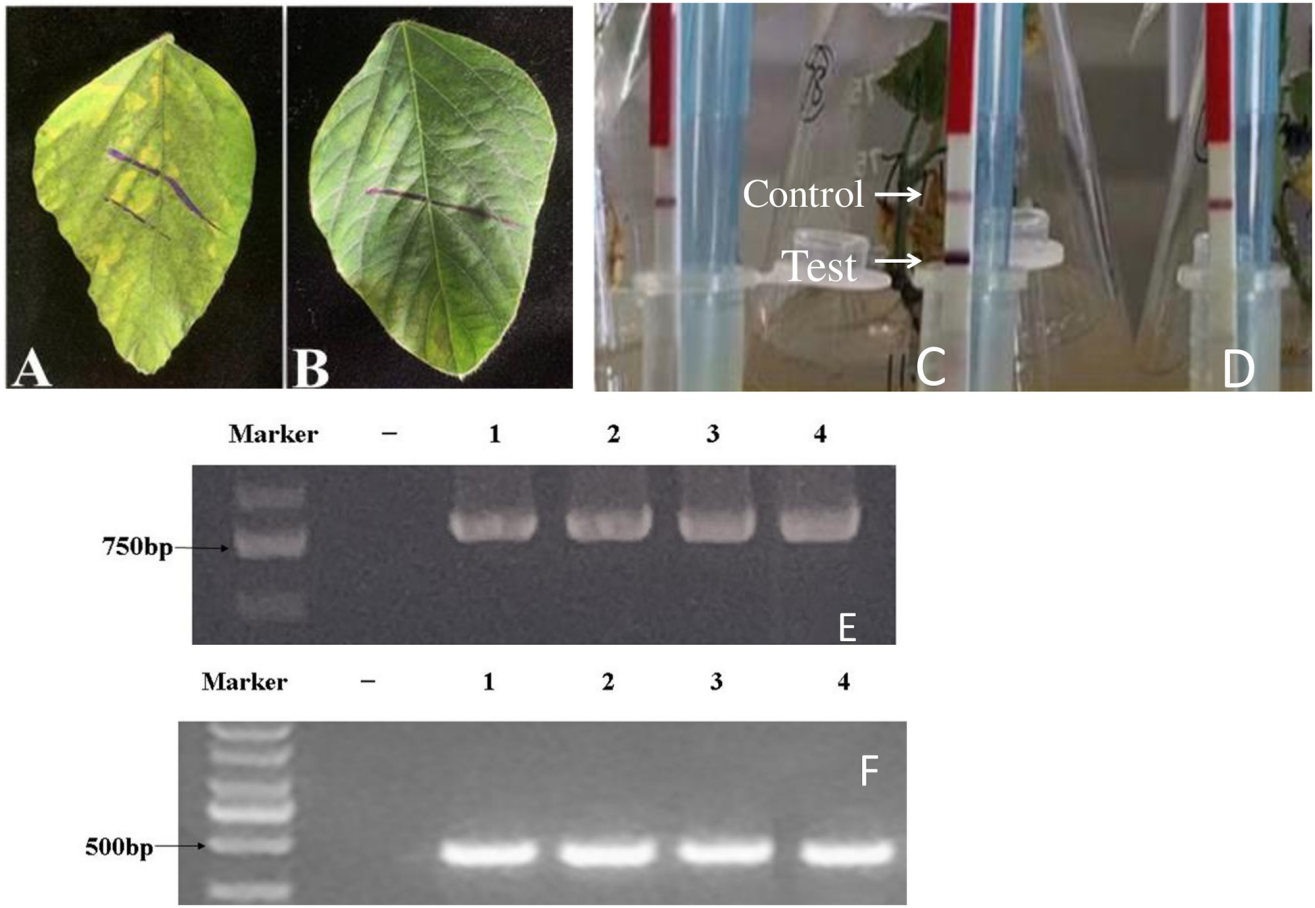
Identification of glufosinate-ammonium herbicide positive plants in transgenic soybean. (A)non-genetically modified Shennong 9. (B)Genetically modified plants. BAR test strips test and identify positive plants. (C)non-genetically modified Shennong 9. (D)Genetically modified negative plants. (E)Agarose gel electrophoresis image of *GmFBX322* gene PCR results of soybean DNA. (F)Soybean DNA BAR gene PCR results agarose gel electrophoresis image.

The results of the BAR test strips of soybean leaves showed that only one control line appeared in the nontransgenic Shennong 9 negative control group, indicating that the wild-type Shennong 9 genome did not contain BAR. The presence of a control line in the transgenic genome indicated that GmFBX322 was not integrated into the soybean genome (Fig 3A and 3B). In the transgenic plants, both a control line and a test line appeared, indicating that the BAR gene, closely linked to GmFBX322, was expressed in the soybean plants and that GmFBX322 was successfully integrated into the soybean genome.” [20, 21] (Fig 3C and 3D).

The DL2000 DNA marker validation band appeared slightly above the 750-bp band, with the amplified fragment size aligning closely with the estimated 771-bp size of GmFBX322. This indicates the successful integration of the gene of interest into the soybean genome. Notably, the marker served as the negative control, and samples 1–4 represented the gene product of the transgenic line (Fig 3E) [22].

Similarly, the DL2000 DNA marker validation band in the agarose gel electrophoresis displayed a clear band slightly below the 500-bp band, corresponding to the estimated position of BAR at 436 bp. This indicates the integration of BAR into the soybean genome. The marker served as the negative control, and samples 1–4 represented the BAR gene product of the transgenic line [22, 23]. Samples 1–4 were the PCR products of the transgenic plant DNA containing GMFBX322 (Fig 3F) [23].

### Salt tolerance identification

**Fig 4:**
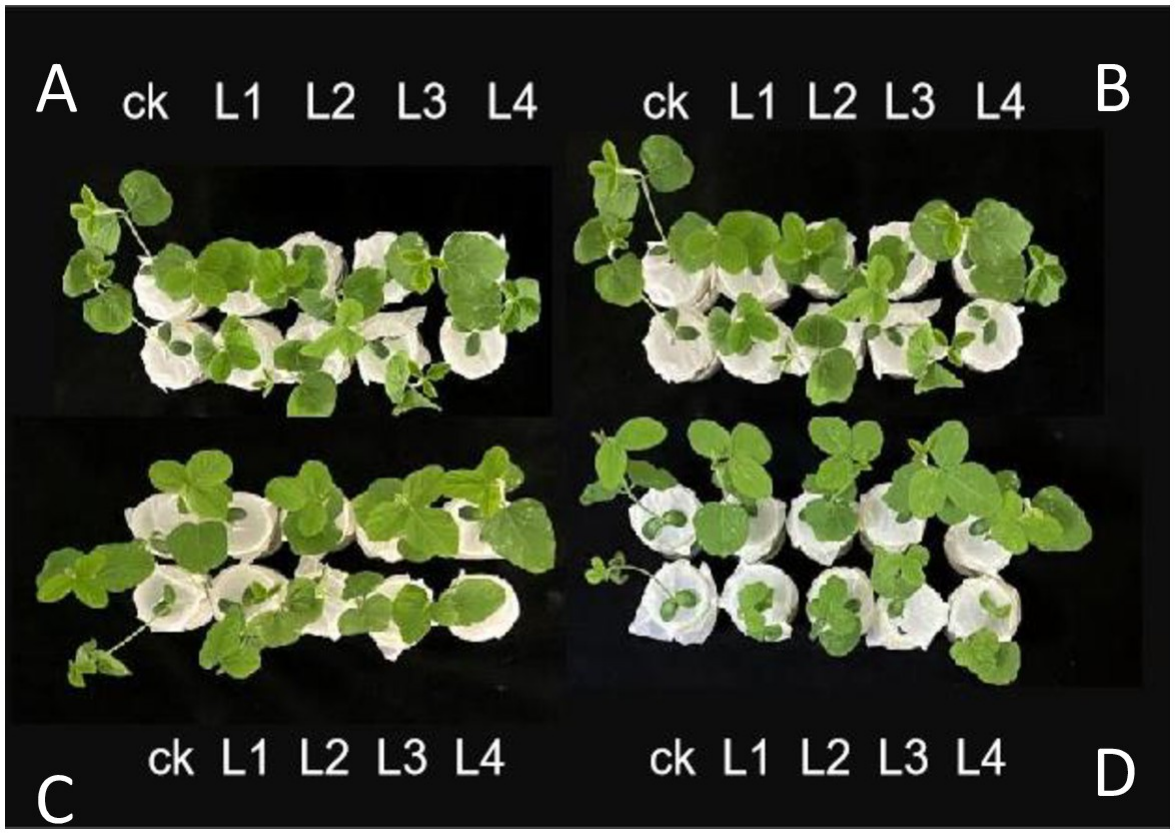
Wild-type soybean plants and overexpression plants after salt stress at different times. A: Day 0; B: Day 1; C: Day 2; D: Day 3; CK: wild-type. Shennong 9 S9 untreated and 150 mM NaCl treated plants; L1: overexpressed lines 2265 untreated and 150 mM NaCl treated plants; L2: overexpressed lines 2267 untreated and 150 mM NaCl treated plants; L3: 2269 untreated and 150 mM NaCl treated plants; L4: overexpressed line 2271 untreated and 150 mM NaCl treated plants.

On the third day of culture, the leaves of the salt-treated plants labeled CK appeared more wilted compared to the previous day when they were cultured with complete Hoagland nutrient solution without NaCl and with 150 mM NaCl, respectively. However, the plants labeled L1, L2, L3, and L4 did not exhibit obvious loss of green color, dehydration, or wilting (Fig 4A). As observed in the figure, the leaves of Shennong 9 continued to wilt progressively over several days following salt treatment, eventually drying out by the third day. Conversely, the transgenic strain continued to grow (Fig 4B–4D).

### GmFBX322 gene expression level detection

**Fig 5:**
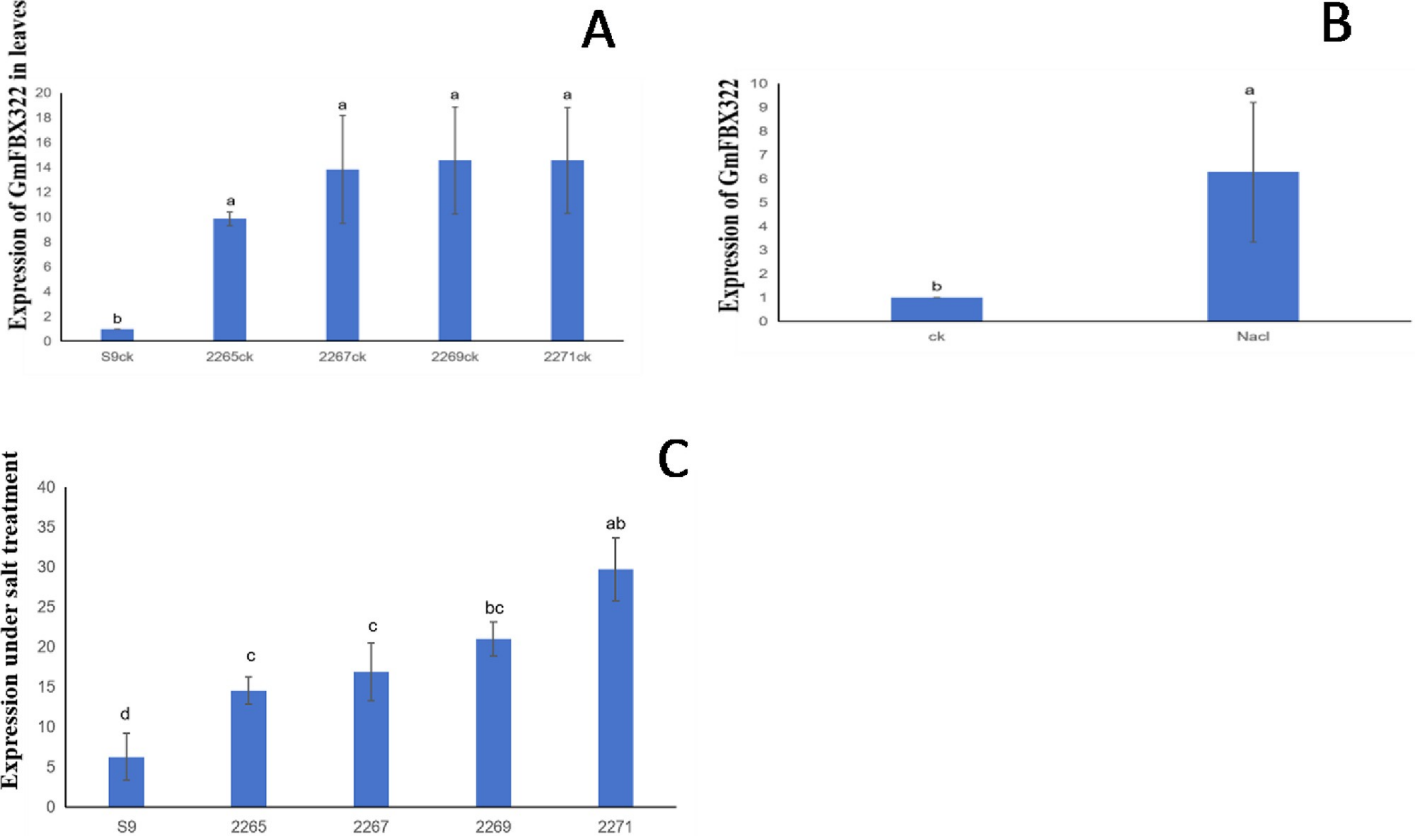
Relative expression levels of GmFBX322 in soybean plant leaves. (A) S9: wild-type Shennong 9; 2265–2267: Overexpression of GmFBX322 soybean lines. The data in the figure are from three biological replicates. (B) CK: Shennong No. 9 soybean plant; NaCl: 150 mM NaCl-treated soybean plants of Shennong 9; The data in the figure are from three biological replicates. (C) S9: wild-type Shennong 9; 2265–2267: Overexpression of GmFBX322 soybean lines. Gene expression of transgenic lines treated with salt. The data in the figure are from three biological replicates.

InTo investigate GmFBX322 expression, qRT-PCR was used to analyze gene expression in soybean leaves without salt treatment. The expression levels of the transgenic lines 2265, 2267, 2269, and 2271 were 9.882321801, 11.82992221, 10.5031674, and 9.399467407, respectively (Fig 5A). The gene expression level of the transgenic strain GmFBX322 differed significantly from that of the wild type.

In the experiment, Shennong 9 plants were treated with salt alone, resulting in a notably higher expression level of GmFBX322 compared with the untreated plants. The expression level wild-type Shennong 9 plants increased by approximately 6.27 times, as observed through qRT-PCR analysis(Fig 5B). These results indicate that salt stress can induce GmFBX322 expression.

To further confirm the salt resistance of the transgenic strain, 150 mM NaCl was used in the experiment. GmFBX322 expression levels in the transgenic strain were measured as 12.22329322, 16.85283889, 24.1892062, and 32.71301614 (Fig 5C) [24].

### Ion concentration expression

**Fig 6:**
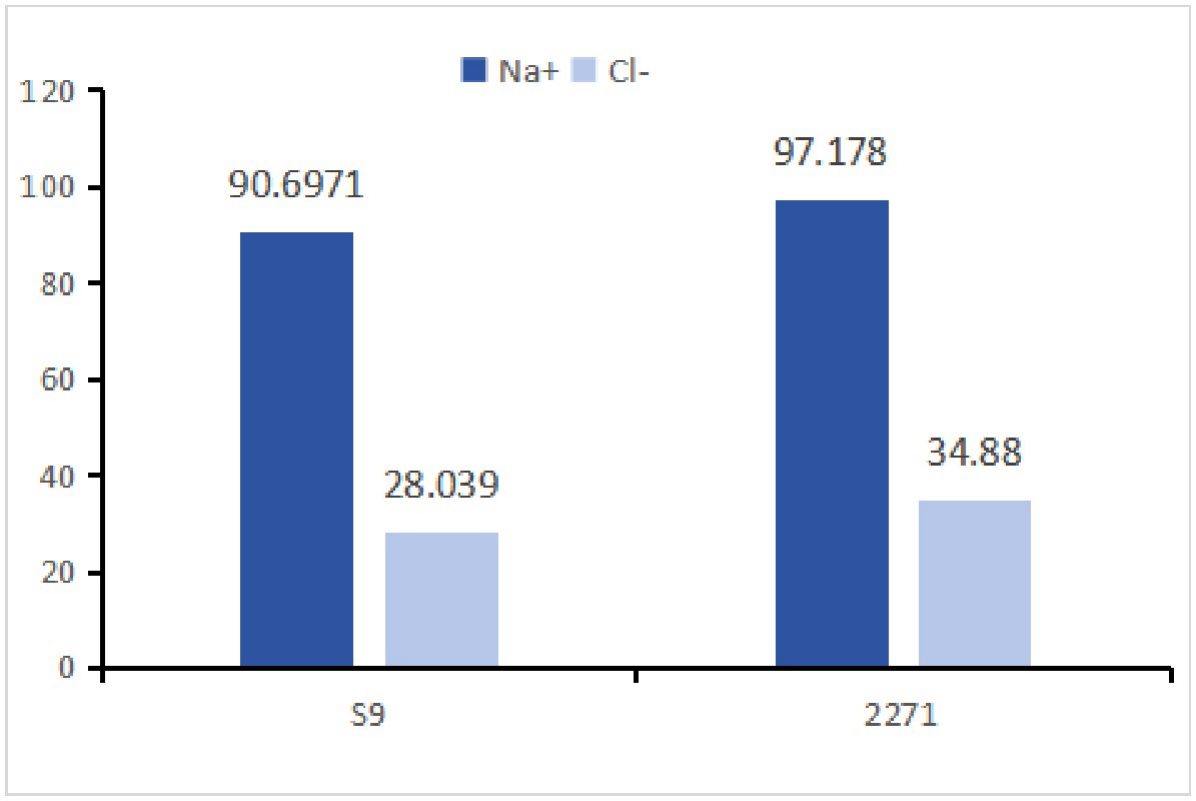
Relative expression levels of GmFBX322 in soybean plant leaves. (A) S9: wild-type Shennong 9; 2265–2267: Overexpression of GmFBX322 soybean lines. The data in the figure are from three biological replicates. (B) CK: Shennong No. 9 soybean plant; NaCl: 150 mM NaCl-treated soybean plants of Shennong 9; The data in the figure are from three biological replicates. (C) S9: wild-type Shennong 9; 2265–2267: Overexpression of GmFBX322 soybean lines. Gene expression of transgenic lines treated with salt. The data in the figure are from three biological replicates.

These soils contain high concentrations of carbonate, chloride, sodium, calcium sulfate, and magnesium sulfate. The most common ions found in these soils are Na^+^, Cl^−^, and SO_4_^2−^, and understanding how these ions affect the physiological and biochemical regulation of plants is crucial to comprehending the undergrowth observed and to gaining deeper insight into the mechanisms of resistance. Na^+^, Cl^−^ and SO_4_^2−^ plasmas have great effects on plant tissues. In this experiment, we measured the concentration of Na^+^ and Cl^−^ in the soil. The integrated Na^+^ and Cl^−^ concentrations in the salt-treated samples were 90.6971 and 28.039, respectively, while in the GM strain, the concentrations were 97.178 and 34.88, respectively (Fig 6). The results indicate that the Na^+^ and Cl^−^ concentrations in the transgenic plants under salt treatment were higher than those in the Shennong 9 plants under the same treatment. This indicates that the salt tolerance of transgenic plants is higher compared to that of Shennong 9 plants [25].

### Identification of salt tolerance in soybeans

**Fig 7:**
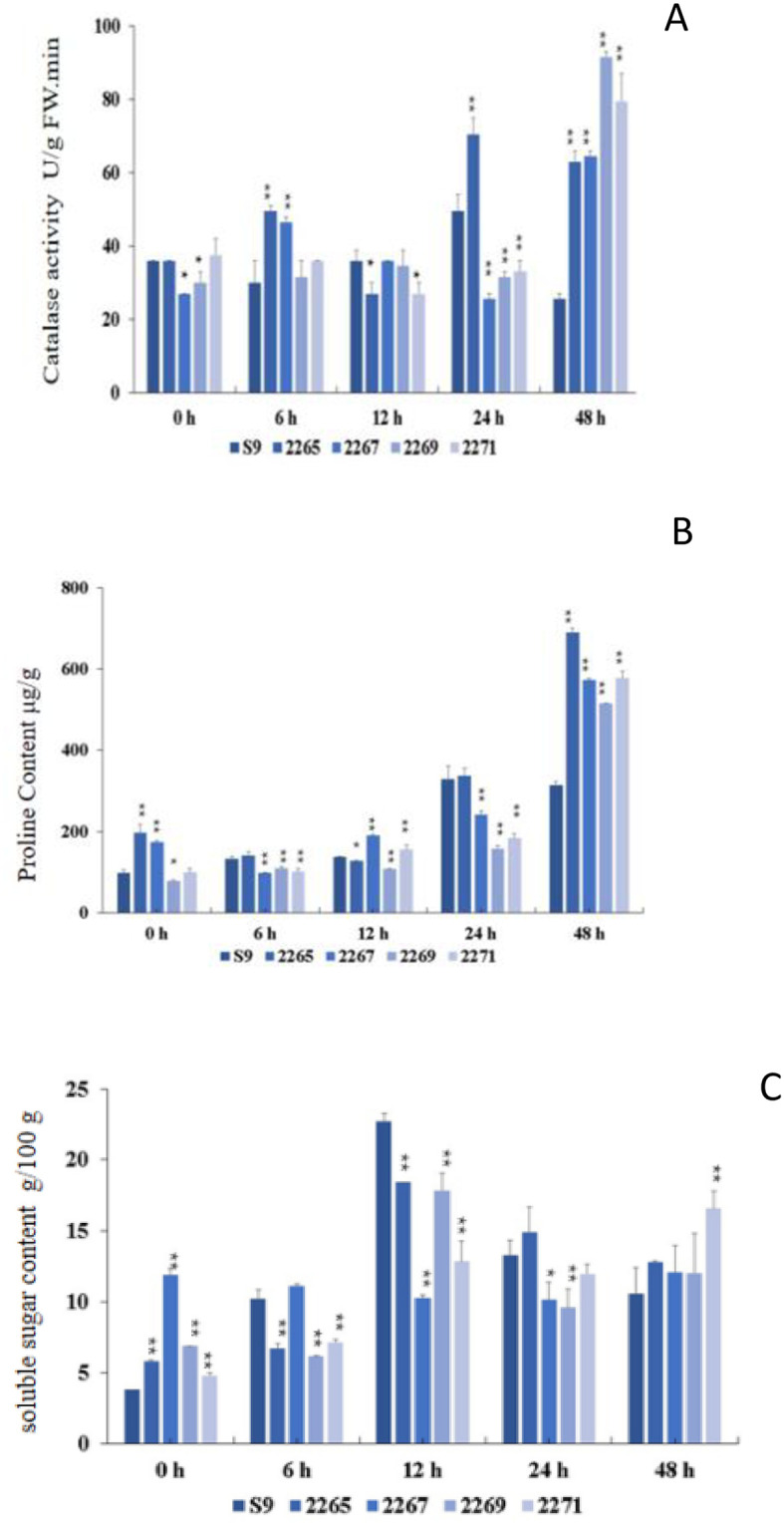
(A)Changes of catalase activity in soybean plant leaves after salt stress. (B) Changes of soluble sugar content in soybean plant leaves after salt stress. (C) Changes of Proline Content in Soybean Plant Leaves after Salt Stress.S9 is Wild-type Shennong 9,2265–2271 are overexpressed lines,* represents a significant difference at.*p = 0*.*05*.

After 48 h of salt treatment, the catalase activity in the leaves of transgenic lines 2265, 2267, 2269, and 2271 was significantly higher than that of wild-type Shennong 9 plants, showing a highly significant correlation. The catalase activity of wild-type Shennong 9 plants was 25.50 U/g FW.min. In comparison, the catalase activity of transgenic line 2265 was 63.00 U/g FW.min (2.47 times higher than the wild type). Transgenic line 2267 had a catalase activity of 64.50 U/g FW.min (2.53 times higher than the wild type). The highest catalase activity was observed in transgenic line 2269, with 91.50 U/g FW.min (3.59 times higher than the wild type). Finally, transgenic line 2271 had a catalase activity of 79.50 U/g FW.min (2.96 times higher than the wild type) (Fig 7A).

The soluble sugar content in the leaves of wild-type Shennong 9 plants first increased and then decreased with extended salt treatment. When not treated with salt, the soluble sugar content in the leaves of the overexpressed lines 2265, 2267, 2269, and 2271 was 5.81 g/100 g, 11.89 g/100 g, 6.86 g/100 g, and 4.78 g/100 g, respectively. These levels were 1.54, 3.15, 1.81, and 1.26 times higher than those in the wild-type plants, which had a soluble sugar content of 3.78 g/100 g. After 48 h of salt treatment, the soluble sugar content in the leaves of the overexpressed lines 2265, 2267, 2269, and 2271 was 12.83 g/100 g, 12.06 g/100 g, 12.05 g/100 g, and 16.59 g/100 g, respectively. This represented increases of 1.22, 1.14, 1.14, and 1.57 times compared with the 10.55 g/100 g content in wild-type plants. The increase in soluble sugar content in the leaves of the overexpressed line 2271 differed significantly from that of the wild-type plants (Fig 7B).

The proline content in the transgenic lines 2265, 2267, and 2271 was 196.58 μg/g, 173.34 μg/g, and 98.99 μg/g, respectively. This was 2.00, 1.76, and 1.01 times higher than that found in wild-type plants, which had a proline content of 98.52 μg/g. Notably, the proline content in the transgenic lines 2265 and 2267 differed significantly from that in the wild-type plants. By the 48th of salt treatment, the proline content in the transgenic lines 2265, 2267, 2269, and 2271 was 689.19 μg/g, 572.08 μg/g, 515.38 μg/g, and 577.19 μg/g, respectively. These values were 2.20, 1.83, 1.65, and 1.84 times higher than that in the wild-type plants, which had a proline content of 313.23 μg/g. The difference in the proline content between the transgenic lines and the wild-type plants was extremely significant (Fig 7C) [26].

## Discussion

The results showed that F-box protein was abundant and expressed in different ways [1], which may play a variety of roles in the process of plant growth and development, and F-box gene played an important role in mediating many different types of stress responses, and F-box protein was involved in plant hormone signaling, which changed the expression of downstream genes by regulating the activity of transcription factors, thereby affecting the stress resistance response of plants [3, 4]. The relative expression levels of DNA in salt solution of FBX homologous genes at different times showed that the expression levels of GmFBX322 and GmFBX254 were relatively large in the heat map, so GmFBX322 with relatively large expressions was selected in this experiment and gene tree analysis showed that GmFBX22 belonged to a typical FBP subfamily. For example, compared with other experiments, this experiment found a more salt-tolerant FBX gene family, and the homologous gene analysis found that GmFBX322 was more salt-tolerant, which provided a certain idea for soybean salt tolerance [20]. As a salt-tolerant soybean variety in global demand, finding salt-tolerant genes and successfully applying them to soybean plants can greatly reduce the low side effects of soybean yield caused by the greenhouse effect and saline-alkali land, and increase yields [21]. The experimental method used the genetic transformation method of soybean cotyledons, and the expression vector carrying GmFBX322 gene was transferred to Shennong 9 soybean cultivar, and the positive plants were identified by three methods: herbicide resistance identification, BAR test strip identification, and molecular detection identification, and the seeds produced by transgenic positive soybean plants were used as materials for follow-up research [25]. The three methods make the results of the experiment more confident and ensure the success of the transgenic gene. The results showed that at the 48th h of salt treatment, the catalase activity of the overexpressed line was more than twice that of the control group Shennong 9. The soluble sugar content was more than 1 times that of Shennong 9 plant in the control group. The proline content was more than 1.5 times that of Shennong 9 plant in the control group.

Three hormones were used to determine that the transgenic plant developed better than the control group, and no auxin, melatonin, cytokinin, abscisic acid and other plant hormones were used [25], because soluble sugar had a greater effect on the growth of leaves, and salt-resistant genes were more expressed on leaves. Proline expression increased during stress, and proline could induce more FBX gene expression [26–28]. In addition, there was no significant difference in agronomic traits between GmFBX322-positive soybean and that of the negative control, which indirectly indicated that this gene did not interfere with the development of other parts of soybean [24, 29]. The results showed that the GmFBX322 gene of transgenic soybean not only provided basic materials for soybean salt tolerance breeding, but also provided scientific research materials and clues for elucidating the mechanism ofFBX gene family [30].

## Supporting information

S1 Fig Agarose gel electrophoresis image of GmFBX322 gene PCR results of soybean DNA. This indicates the successful integration of the gene of interest into the soybean genome.
(TIF)

S2 Fig Soybean DNA BAR gene PCR results agarose gel electrophoresis image. This indicates the integration of BAR into the soybean genome.
(TIF)

S3 Fig Sodium chloride ion concentration in plants. The peak concentration of sodium chloride in the plants was measured, and the salt tolerance of the transgenic lines could be determined to be higher than that of shennong 9.
(PDF)

S1 Table qRT -PCR expression of GmFBX322 gene. The raw data of the five genes of qRT-PCRcontained three biological replicates.
(PDF)

S2 Table The expression of three hormones. Raw hormone data from transgenic strains.
(PDF)
