# Supplementary figures and images for: Genetic transformation of GmFBX322 gene and salt tolerance physiology in soybean
Source: PLoS One. 2024 Sep 12;19(9):e0307706. doi: 10.1371/journal.pone.0307706 (PMC11392233; doi:10.1371/journal.pone.0307706)

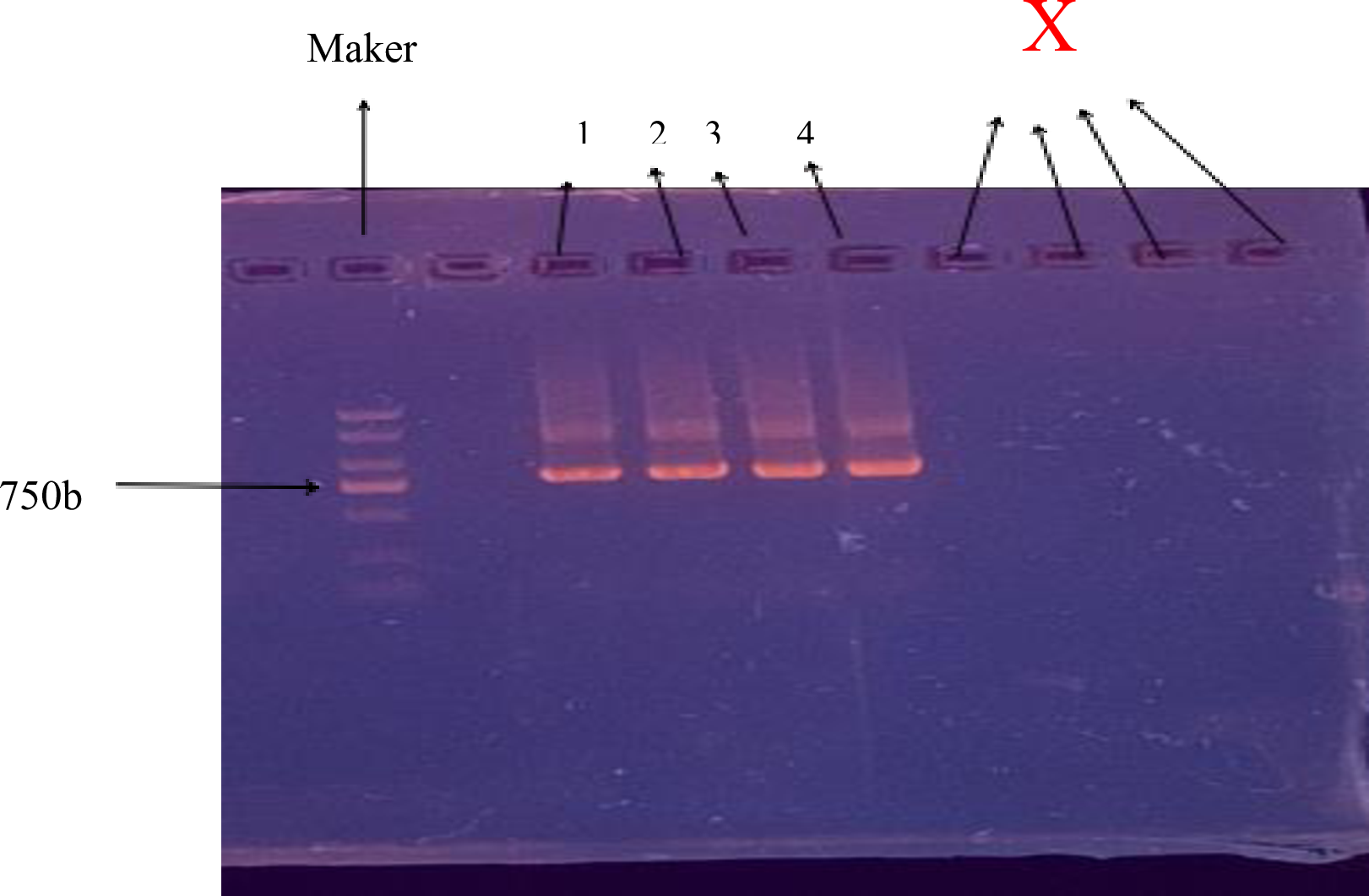

Supplement: S1 Fig — This indicates the successful integration of the gene of interest into the soybean genome. (TIF) [file pone.0307706.s001.tif]

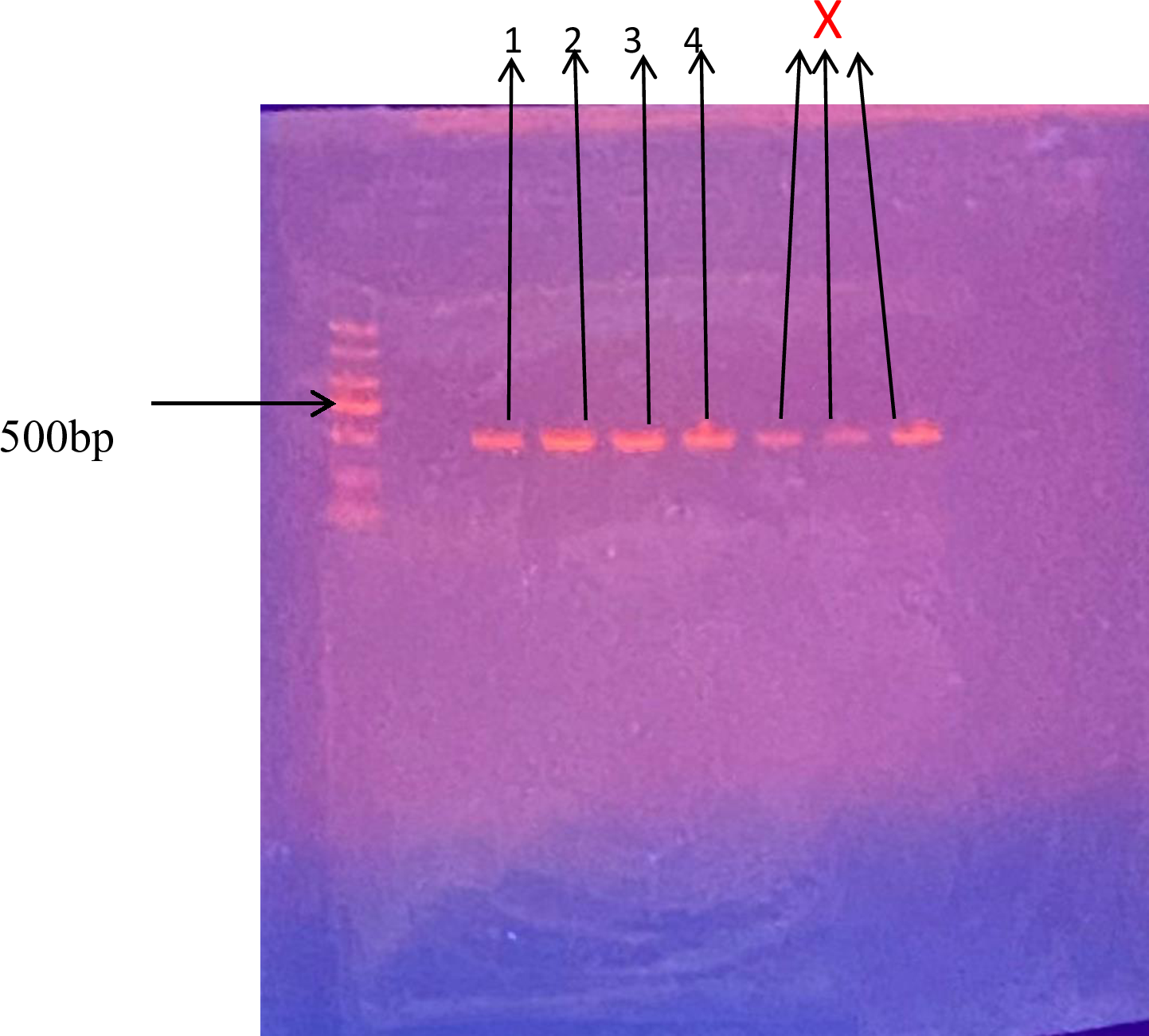

Supplement: S2 Fig — This indicates the integration of BAR into the soybean genome. (TIF) [file pone.0307706.s002.tif]
