# Supplementary material for: Genetic transformation of GmFBX322 gene and salt tolerance physiology in soybean
Source: PLoS One. 2024 Sep 12;19(9):e0307706. doi: 10.1371/journal.pone.0307706 (PMC11392233; doi:10.1371/journal.pone.0307706)

Na^{+}concentration					
1（S9）	90.70				
2(2271)	97.18				

序号	保留时间 min	峰名称	峰类型	峰面积 μS*min	峰高 μS	样品量
2	6.76	Na	MB	0.520	1.984	90.6971
		总计：		0.52	1.98	90.70

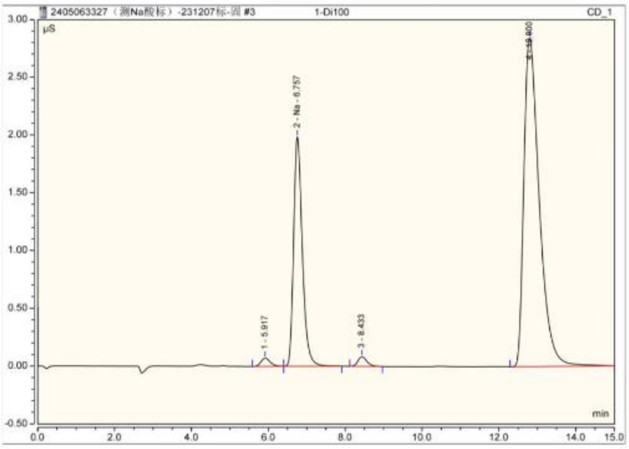

序号	保留时间 min	峰名称	峰类型	峰面积 μS*min	峰高 μS	样品量
2	6.76	Na	BMB	0.557	2.116	97.1787
		总计：		0.56	2.12	97.18

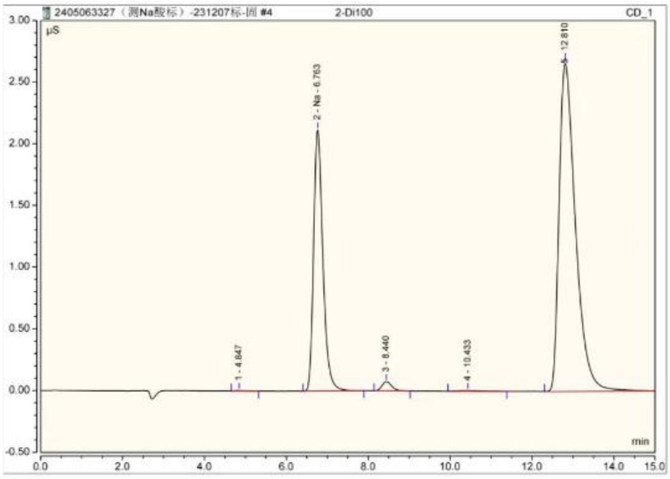

Cl^-vconcentration		
1(S9)	28.04	
2(2271)	34.88	

序号	保留时间 min	峰名称	峰类型	峰面积 μS*min	峰高 μS	样品量
1	3.67	Cl	BM	1.043	10.270	28.0393
		总计:		1.04	10.27	28.04

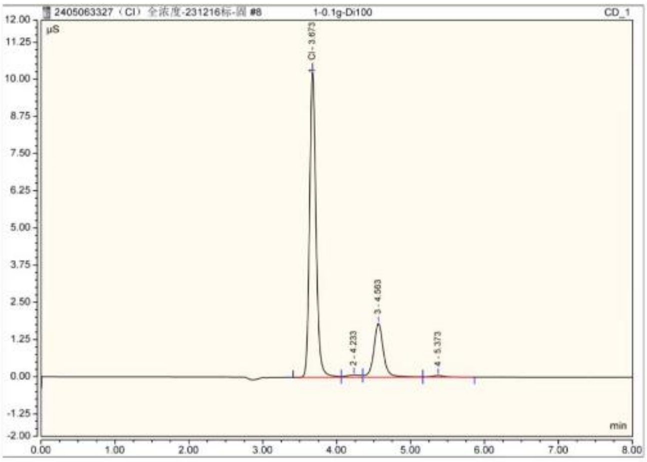

序号	保留时间 min	峰名称	峰类型	峰面积 μS*min	峰高 μS	样品量
1	3.66	Cl	BM	1.300	12.741	34.8813
		总计:		1.30	12.74	34.88

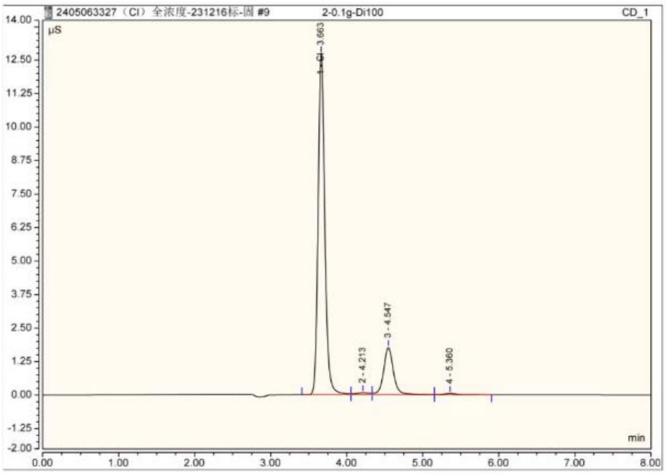

Supplement: S3 Fig — The peak concentration of sodium chloride in the plants was measured, and the salt tolerance of the transgenic lines could be determined to be higher than that of shennong 9. (PDF) [file pone.0307706.s003.pdf]
