# Supplementary material for: Genetic transformation of GmFBX322 gene and salt tolerance physiology in soybean
Source: PLoS One. 2024 Sep 12;19(9):e0307706. doi: 10.1371/journal.pone.0307706 (PMC11392233; doi:10.1371/journal.pone.0307706)
Supplement: S1 Table — The raw data of the five genes of qRT-PCRcontained three biological replicates. (PDF) [file pone.0307706.s004.pdf]

	Ct Brach (The first time)	Ct Actb (The first time)	
S9ck	24.01（22.75/24.31/24.97）	20.58(21.90/19.44/20.4)	
2265ck	24.97(22.45/26.44/26.02)	24.88(26.31/24.74/23.59)	10.12605275
2267ck	24.9(/26.62/24.14/23.94)	25.7(25.69/24.33/27.08)	18.76535919
2269ck	23.73(23.86/24.46/22.87)	23.66（20.29/24.96/25.73）	10.26740718
2271ck	23.29(21.81/23.54/24.52)	23.24(22.25/25.74/21.73)	10.41073484
Salt treatment			
S9 s	23.33(21.79/23.17/25.03)	21.48(23.89/19.77/20.78)	2.989698497
2265 s	22.75(25.11/20.49/22.69)	22.97(23.7/21.92/23.29)	12.60566218
2267 s	22.09(23.53/21.19/21.55)	22.66(24.64/22.72/20.62)	16
2269 s	22.94(23.81/23.88/21.13)	24.93(27.29/22.02/25.48)	42.81368175
2271 s	21.88(23.01/23.12/19.51)	23.32(22.74/24.56/22.66)	29.24260641

Three technical replicates were performed for each set of data and [illegible]

Ct Brach (Second time)	Ct Actb (Second time)	
24.98(22.76/25.12/27.06)	19.77(21.88/19.43/18.01)	
23.84（24.41/24.00/23.11）	21.99(22.94/21.97/21.06)	10.26740718
23.91(22.01/24.30/25.42)	21.29(23.12/21.78/18.97)	6.02098699
24.39(23.87/25.11/25.19)	22.74(20.29/24.14/23.79)	11.79415374
23.78(23.97/22.60/24.77)	21.81(24.27/21.81/19.35)	9.447941291
27.05(27.54/26.31/27.30)	24.95(24.83/26.49/23.53)	8.633825892
22.73(25.89/20.44/21.86)	21.00(18.76/24.79/19.45)	11.15794933
22.91(19.96/26.01/22.76)	22.08(22.73/22.12/21.39)	20.82146969
24.87(27.03/22.00/25.58)	23.56(24,55/22.91/23.22)	14.92852786
22.97(21.85/23.47/23.59)	23.46(25.03/22.76/22.59)	51.98415337

three biological replicates were performed for each set of trea

Ct Brach (The third time)	Ct Actb (The third time)		
24.3(21.66/24.38/26.86)	20.43(20.93/22.11/18.25)		
22.75(24.93/20.33/22.99)	22.09(19.71/21.97/24.59)	9.253505471	9.882321801
22.08(21.19/24.07/20.98)	21.63(21.79/20.66/22.44)	10.70342044	11.82992221
24.31(22.96/24.36/25.61)	23.68(25.31/23.82/21.91)	9.447941291	10.5031674
24.07(25.62/23.33/23.26)	23.26(23.12/21.48/25.18)	8.339726087	9.399467407
23.71(24.72/21.99/24.42)	22.69(23.54/22.41/22.12)	7.210003701	6.277842697
24.16(23.18/26.10/23.50)	23.98(27.05/21.18/23.71)	12.90626815	12.22329322
25.09(26.77/24.41/24.09)	25.00（26.81/24.79/23.40）	13.73704698	16.85283889
23.62(24.22/24.21/22.43)	23.64(25.11/22.68/23.13)	14.82540899	24.1892062
24.12(20.84/25.66/25.86)	24.33(24.21/24.12/24.66)	16.91228865	32.71301614

tments
