# Supplementary material for: Genetic transformation of GmFBX322 gene and salt tolerance physiology in soybean
Source: PLoS One. 2024 Sep 12;19(9):e0307706. doi: 10.1371/journal.pone.0307706 (PMC11392233; doi:10.1371/journal.pone.0307706)
Supplement: S2 Table — Raw hormone data from transgenic strains. (PDF) [file pone.0307706.s005.pdf]

	0h	6h	12h	24h	48h
	36	36	39	45	27
S9	36	24	33	54	24
	36	30	36	49.5	25.5
	36	51	24	75	66
2265	36	48	30	66	60
	36	49.5	27	70.5	63
	27	45	36	27	63
2267	27	48	36	24	66
	27	46.5	36	25.5	64.5
	33	27	39	33	93
2269	27	36	30	30	90
	30	31.5	34.5	31.5	91.5
	33	36	30	36	87
2271	42	36	24	30	72
	37.5	36	27	33	79.5

		0h	6h	12h	24h	48h
S9		3.795	10.86	22.11	12.21	10.92
		3.765	9.54	23.265	14.355	10.17
		3.78	10.2	22.6875	13.2825	10.545
	2265	5.76	7.035	18.39	13.17	12.765
		5.865	6.405	18.405	16.725	12.885
		5.8125	6.72	18.3975	14.9475	12.825
	2267	11.4	11.265	10.47	11.4	13.98
		12.375	11.025	10.05	8.865	10.14
		11.8875	11.145	10.26	10.1325	12.06
	2269	6.885	6.21	16.65	10.92	9.27
		6.84	6.18	19.05	8.265	14.835
		6.8625	6.195	17.85	9.5925	12.0525
	2271	4.56	7.35	11.37	12.675	15.405
		4.995	6.945	14.31	11.265	17.775
		4.7775	7.1475	12.84	11.97	16.59

		0h	6h	12h	24h	48h
S9		105.4928	125.0113	138.9531	296.0305	303.4661
		91.55103	138.9531	135.2353	361.0921	322.9846
		98.52192	131.9822	137.0942	328.5613	313.2253
	2265	217.0271	149.1771	125.0113	356.4449	701.2716
		176.1312	131.5175	128.7291	317.4079	677.1058
		196.5791	140.3473	126.8702	336.9264	689.1887
	2267	177.0606	96.19829	187.2846	233.7572	566.501
		169.625	98.98664	191.9318	250.4873	577.6545
		173.3428	97.59247	189.6082	242.1223	572.0777
	2269	74.82089	112.9284	108.2812	151.036	514.4517
		81.32705	107.3517	108.2812	164.0483	516.3106
		78.07397	110.1401	108.2812	157.5421	515.3812
	2271	108.2812	108.2812	144.5298	172.4134	559.9949
		89.69212	92.48048	165.9072	193.7908	594.3846
		98.98664	100.3808	155.2185	183.1021	577.1897
